# Frequency and risk factors for incident and redetected *Chlamydia trachomatis* infection in sexually active, young, multi-ethnic women: a community based cohort study

**DOI:** 10.1136/sextrans-2014-051607

**Published:** 2014-08-06

**Authors:** Adamma Aghaizu, Fiona Reid, Sally Kerry, Phillip E Hay, Harry Mallinson, Jorgen S Jensen, Sarah Kerry, Sheila Kerry, Pippa Oakeshott

**Affiliations:** 1 Division of Population Health Sciences, St George's, University of London, London, UK; 2 HIV & STI Department, Centre for Infectious Disease Surveillance and Control, Public Health England, London, UK; 3 Barts and the London, School of Medicine and Dentistry, Queen Mary, University of London, London, UK; 4 Department of Genitourinary Medicine, St George's Hospital, London, UK; 5 Aintree Hospital, Liverpool, UK; 6 Statens Serum Institut, Copenhagen, Denmark

**Keywords:** Chlamydia Infection, Chlamydia Trachomatis, Public Health, Sexual Health, Testing

## Abstract

**Objective:**

To investigate the frequency and risk factors for incident and redetected *Chlamydia trachomatis* infection in sexually active, young, multi-ethnic women in the community.

**Design:**

Cohort study.

**Setting:**

20 London universities and Further Education colleges.

**Participants:**

954 sexually experienced women, mean age 21.5 years (range 16–27), 26% from ethnic minorities, who were recruited to the Prevention of Pelvic Infection (POPI) chlamydia screening trial between 2004 and 2006, and returned repeat postal self-taken vaginal swabs 11–32 (median 16) months after recruitment.

**Results:**

The estimated annual incidence of chlamydia infection among 907 women who tested negative at baseline was 3.4 per 100 person-years (95% CI 2.5 to 4.6 per 100 person-years), but 6.6 per 100 person-years (95% CI 4.5 to 9.3 per 100 person-years) in the 326 teenagers (<20 years). Predictors of incident chlamydia infection were age <20 years (relative risk (RR) 4.0, 95% CI 2.1 to 7.5), and (after adjusting for age) a new sexual partner during 12 months follow-up (RR 4.4, 95% CI 2.0 to 9.9), smoking (RR 2.2 95% CI 1.2 to 3.9), concurrent bacterial vaginosis (RR 2.0 95% CI 1.1 to 3.9) and high risk carcinogenic human papillomavirus (RR 2.2, 95% CI 1.1 to 4.3). Of 47 women positive for chlamydia at baseline, 12 (25.5%, 95% CI 13.9% to 40.3%) had redetected infection at a median of 16 months follow-up. Taking into account follow-up time (65 person-years), the annual redetection rate was 18.5 per 100 person-years (95% CI 9.9 to 30.0 per 100 person-years).

**Conclusions:**

One in four women with chlamydia infection at baseline retested positive, supporting recent recommendations to routinely retest chlamydia positives.

## Introduction

With the English National Chlamydia Screening Programme (NCSP) completing its 10th year in 2013, much focus has been on the effectiveness of this and similar programmes in the USA and Europe for reaching their aims of controlling chlamydia infection through opportunistic screening. English NCSP guidelines recommend retesting annually or with every new partner for all 16–24 year olds and, in 2013, began to include recommending retesting for those found to be positive.^1^ In 2012, at 25.8% coverage of 15–24 year olds, 1 782 122 tests were undertaken in England with a positivity rate of 7.7%.^2^ However, recent UK data from the National Survey of Attitudes and Lifestyles show little change in the population prevalence of chlamydia in women in this age group: 3.2% in 2010 versus 3.1% in 2000.^3^

It is difficult to measure the incidence and reinfection rates of *Chlamydia trachomatis* in women as it is usually asymptomatic and relies on affected persons seeking a test. Consequently, there are few British published data on the incidence and reinfection rates of chlamydia^4–6^ and none in women recruited outside healthcare facilities. We examined frequency and risk factors for incident and redetected chlamydia infection in women who provided follow-up samples in the Prevention of Pelvic Infection (POPI) chlamydia screening trial.^7^ ^8^

## Methods

### Participants and setting

The design, recruitment methods and participants of the POPI trial have been described elsewhere.^7–10^ Briefly, between 2004 and 2006, 2529 sexually active female students were recruited from London universities and Further Education colleges to a chlamydia screening trial. Participants were eligible if they were aged ≤27 years, sexually active, not pregnant and had not been tested for chlamydia in the previous 3 months. At baseline, they completed a questionnaire on socio-demographic characteristics and sexual behaviour and provided two self-taken vaginal specimens. One was used for the chlamydia screening trial. The other was rolled over a glass slide for analysis for bacterial vaginosis, placed in Aptima transport medium and stored at -80°C for later testing for *Mycoplasma genitalium* (by an inhibitor controlled PCR detecting the 16S ribosomal gene)^9^ and human papillomavirus (by Digene Hybrid Capture 2 assay and the Roche Linear Array Genotyping assay).^10^ Of 2529 participants, 94% (2377) were followed up after 12 months by questionnaire and/or medical records search, and 38% (954) also returned a repeat postal sample 11–32 (median 16) months after recruitment. As with baseline samples these were tested for *C. trachomatis* using TMA (Gen-Probe Inc). For the 12 women with redetection of *C. trachomatis*, typing was attempted using the *omp1* typing system described by Jurstrand *et al*.^11^

### Statistical methods

The analyses for the current study were restricted to 954 participants who returned self-taken repeat postal specimens. We analysed the intervention and control groups combined as all women with chlamydia infection at baseline were referred for treatment either shortly after recruitment (intervention group) or after 12 months (deferred screening control group), and repeat postal samples were provided later, a median of 16 months after recruitment (except in one case where the sample was returned after only 11 months). As recommended at recruitment, 25% (235/952) of participants got themselves tested for chlamydia independent of the trial during follow-up, and this was equally distributed between the intervention (123/499) and control groups (112/453). We investigated the prevalence of chlamydia at baseline, and the rate of incident and redetected infections at follow-up. Infection in both baseline and follow-up samples was classified as redetection, recognising that these cases may either be due to clearance (including clearance following treatment) and subsequent reinfection, or persistent infection. However, the majority of redetected cases are likely to be reinfections, since all women with chlamydia at baseline were ultimately referred for treatment,^8^ and around half of any untreated infections would be expected to resolve spontaneously within the year.^12^

We conducted exploratory analyses investigating demographic and behavioural risk factors for incident and redetected chlamydia infections, and estimated relative risks (RRs) using binomial regression (StataCorp 2011, Stata Statistical Software: Release 12. College Station, Texas, USA: StataCorp LP). Analyses of predictors for incident infection were adjusted for age, as young age is a known risk factor for chlamydia. Due to the small number of redetected infections, we only examined the five risk factors which were shown to be independently associated with incident infection.

We used the dates of the baseline and repeat samples as the beginning and end dates of follow-up. Annual incidence rates were estimated by dividing the number of observed new cases by the total person-years followed up, for subjects negative at baseline. Annual redetection rates were estimated by dividing the number of redetected cases by the total person-years followed up, for subjects positive at baseline. We then expressed these as rates per 100 person-years. Calculation of the true incidence or redetection rates would have required information on the date of infection which was not available.

## Results

### Participant characteristics

The mean age of the 954 participants who returned repeat samples was 21.5 years (SD 2.9, range 16–27 years) and 26% (251) were of an ethnic minority background (black African n=94, black Caribbean n=74, black other n=12 and other ethnic groups n=71). In all, 77% (737/954) were recruited from universities and the remainder from FE colleges; 37% (352/954) were teenagers aged <20 years.

Table 1 shows that women who returned repeat postal specimens were similar to those who did not in the proportion who reported a new partner in the previous year and who were aged <16 years at first sex. However, they were slightly older, and less likely to be of black ethnicity or to have had chlamydia or bacterial vaginosis at baseline.

**Table 1:** Baseline characteristics of 2519* female students who did (n=954) or did not (n=1565) provide repeat postal samples a median of 16 months after recruitment

Characteristics at baseline	% (n/N) of 1565 women who did not return follow-up samples	% (n/N) of 954 women who returned follow-up samples
Age <20 years	49.1 (769/1565)	36.9 (352/954)
Black ethnicity	32.6 (505/1550)	18.9 (180/952)
Smoker	34.5 (536/1556)	26.9 (256/952)
New partner in the previous year	48.8 (509/1044)	48.8 (465/953)
Age <16 at first sex	29.7 (453/1525)	29.3 (277/945)
Oral contraception	43.7 (673/1541)	54.5 (518/951)
Uses condoms	58.3 (900/1542)	47.5 (452/951)
*Chlamydia trachomatis*	6.1 (96/1565)	4.9 (47/954)
Previous history of *C. trachomatis*	5.4 (81/1492)	7.5 (68/908)
*Mycoplasma genitalium*	3.5 (51/1470)	2.9 (26/907)
Bacterial vaginosis	27.8 (333/1463)	17.1 (156/914)
*Neisseria gonorrhoeae*	0.5 (7/1483)	0.2 (2/914)

*Ten of the total 2529 women did not provide adequate samples for chlamydia testing at baseline.

### Incidence of chlamydia infection

**Figure 1:**
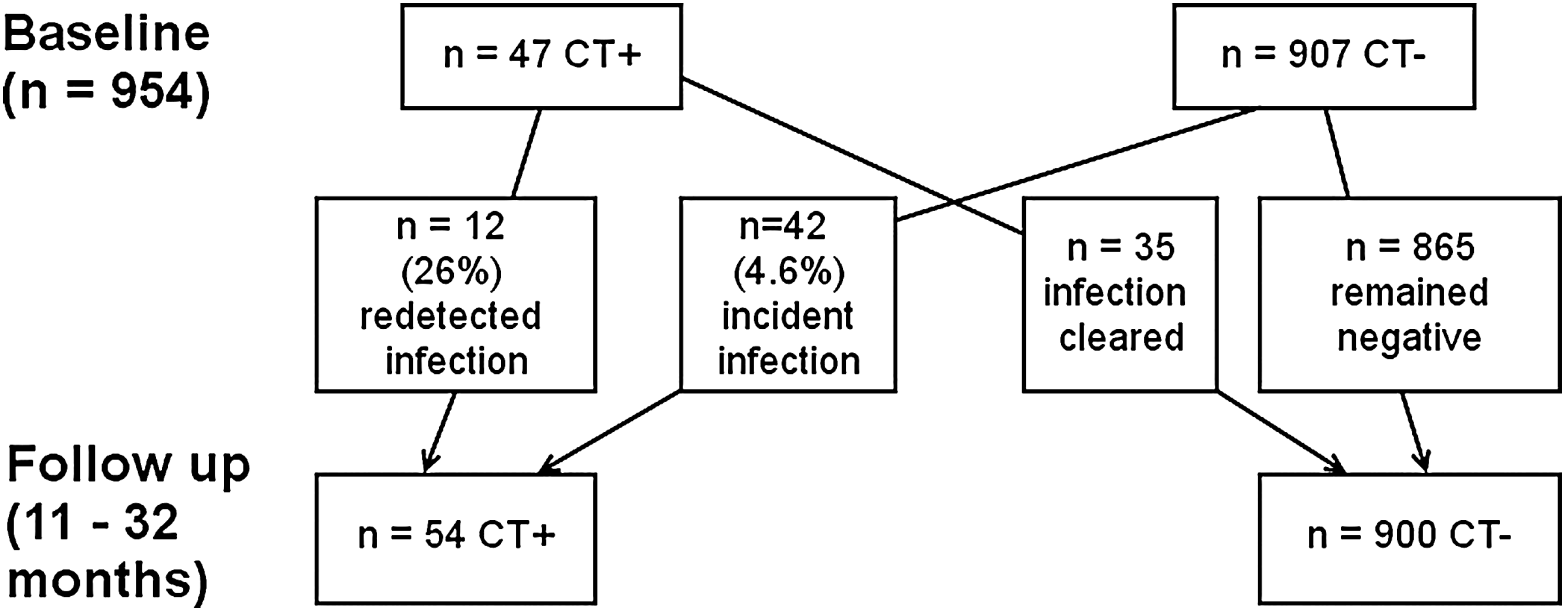
Flowchart for 954 women who provided repeat postal samples 11–32 (median 16) months after recruitment.

Among 907 women who were negative for chlamydia at baseline and followed up for 11–32 (median 16) months, the proportion with incident chlamydia infection was 4.6% (n=42, 95% CI 3.4% to 6.2%) (figure 1). Taking into account the total follow-up time (1234 person-years), the estimated annual incidence of infection was 3.4 per 100 person-years (95% CI 2.5 to 4.6 per 100 person-years). In participants aged <20 years, the proportion with incident infection was 8.9% (29/326, 95% CI 6.0% to 12.5%) and the annual rate was 6.6 per 100 person-years (95% CI 4.5 to 9.3 per 100 person-years). Predictors of incident chlamydia infection were age <20 years (RR 4.0, 95% CI 2.1 to 7.5), and (after adjusting for age) a new sexual partner during 12 months follow-up (RR 4.4, 95% CI 2.0 to 9.9), smoking (RR 2.2, 95% CI 1.2 to 3.9), baseline bacterial vaginosis (RR 2.0, 95% CI 1.1 to 3.9) and baseline high risk carcinogenic human papillomavirus (RR 2.2, 95% CI 1.1 to 4.3) (table 2).

**Table 2:** Predictors of incident chlamydia infection (n=42) in 907 women who were chlamydia negative at baseline and provided repeat postal samples after 11–32 months

	Percentage of women with characteristic	Incidence of chlamydia % (proportion) of women		Relative risk (95% CI)	Adjusted relative risk* (95% CI)
Characteristic		With characteristic	Without characteristic		
Age <20 years	35.9	8.9 (29/326)	2.2 (13/581)	4.0 (2.1 to 7.5)	–
Black ethnicity	17.5	5.7 (9/158)	4.4 (33/747)	1.3 (0.6 to 2.6)	1.1 (0.5 to 2.2)
Smoker	27.1	7.8 (19/245)	3.5 (23/660)	2.2 (1.2 to 4.0)	2.2 (1.2 to 3.9)
New partner during 12 months follow-up	48.0	8.0 (35/435)	1.5 (7/471)	5.4 (2.4 to 12.0)	4.4 (2.0 to 9.9)
Condom use during 12 months follow-up†	55.0	5.5 (27/487)	3.8 (15/399)	1.5 (0.8 to 2.7)	1.2 (0.6 to 2.1)
Age at first sex <16 years	29.3	6.8 (18/263)	3.8 (24/636)	1.8 (1.0 to 3.3)	1.5 (0.8 to 2.7)
*Neisseria gonorrhoeae*‡	0.2	0 (0/2)	4.7 (41/867)	–	–
Bacterial vaginosis‡	16.2	8.5 (12/141)	3.7 (27/727)	2.3 (1.2 to 4.4)	2.0 (1.1 to 3.9)
*Mycoplasma genitalium*‡	2.9	12.0 (3/25)	4.3 (36/837)	2.8 (0.9 to 8.5)	2.8 (2.3 to 9.0)
Carcinogenic HPV‡	16.4	8.5 (11/130)	3.8 (25/663)	2.2 (1.1 to 4.5)	2.2 (1.1 to 4.3)

*Adjusted for age <20 years.

†No data available on consistency of use.

‡At baseline.

### Redetection rates of chlamydia infection

Among the 954 women providing follow-up samples, 4.9% (n=47, 95% CI 3.6% to 6.5%) tested positive for chlamydia at recruitment and 5.7% (n=54, 95% CI 4.3% to 7.3%) were positive at follow-up. The proportion with redetected chlamydia among 47 women positive at recruitment was 25.5% (n=12, 95% CI 13.9% to 40.3%). Taking into account the total follow-up time of 65 person-years, the annual redetection rate was 18.5 per 100 person-years (95% CI 9.9 to 30.0 per 100 person-years). Chlamydia redetection was not significantly associated with any of the tested risk factors, but numbers were small (table 3).

**Table 3:** Predictors of redetected chlamydia infection (n=12) after 11–22 months in 47 women who were chlamydia positive at baseline*

Characteristic	Percentage of women with characteristic	Redetected infection with chlamydia % (proportion) of women		Relative risk (95% CI)
		With characteristic	Without characteristic	
Age <20 years	55.3	26.9 (7/26)	23.8 (5/21)	1.13 (0.42 to 3.05)
Smoking	23.4	9.0 (1/11)	30.6 (11/36)	0.30 (0.04 to 2.06)
New partner during 12 months follow-up	63.8	20.0 (6/30)	35.3 (6/17)	0.57 (0.22 to 1.48)
Bacterial vaginosis†	32.6	26.7 (4/15)	25.8 (8/31)	1.03 (0.37 to 2.89)
Carcinogenic HPV†	40.0	6.3 (1/16)	41.7 (10/24)	0.15 (0.02 to 1.06)

*Of the 47 chlamydia positives at baseline, 17 were in the intervention group and 30 in the deferred screening control group. Of the 12 redetected cases of chlamydia infection at follow-up, three were in the intervention group and nine in the deferred screening control group.

†At baseline.

Among the 12 women with redetection of chlamydia infection, six had baseline and follow-up specimens with adequate DNA load for genetic typing. Four had the same *omp1* genotype detected in both specimens suggesting treatment failure, reinfection from an untreated partner or reinfection from a new partner carrying the same genotype. Two, however, were probably infected from a new partner, as the genotype of the *C. trachomatis* strain had changed between the two time points.

## Discussion

### Principal findings

In this community based cohort, the annual incidence of chlamydia infection was 3.4 per 100 person-years. Predictors of incident chlamydia infection were age <20 years, a new partner in the previous year, smoking, and concurrent bacterial vaginosis or high risk human papillomavirus. One in four women with chlamydia at baseline tested positive again after a median of 16 months. Taking into account the total follow-up time, the estimated annual redetection rate was 18.5 per 100 person-years.

### Strengths and weaknesses

This is the first study to examine incident and redetected chlamydia rates in a community sample of sexually active female students. It provides much needed data on the rates of new infections and the characteristics of those most affected. It includes a large number of participants from ethnic minority backgrounds and teenagers, who are often hard to reach. It was a pragmatic study, where women diagnosed with an infection were able to choose where and when to be treated. It involved the use of self-taken vaginal samples, which are used in the English NCSP and have a higher sensitivity for the detection of chlamydia than urine samples.^13^ A higher follow-up rate was achieved than reported for some other home sampling studies.^14^ ^15^ In addition, we had data on smoking, age at first sexual intercourse and rates of co-infections with other STIs. *M. genitalium* may be a surrogate marker for a high number of sexual partners.^9^ Although numbers were small, genetic typing suggested that 2/6 redetected chlamydia infections were due to a new sexual partner which is in line with previous reports.^16^ ^17^

A limitation is that incidence rates are likely to have been underestimated. First, women may have acquired and cleared an infection in the time between baseline and follow-up. The average duration of untreated chlamydia infection is unknown; data suggest that most infections remain for over 60 days and some may persist for years.^12^ ^18^ Second, one in four women reported that they had had an independent test for chlamydia outside of the study during the follow-up period and may have had an infection diagnosed and treated. Third, only 38% of the cohort provided samples at follow-up, and these women were slightly older, less likely to be of black ethnicity and had lower rates of STIs at baseline, indicating that these were lower risk women compared with non-responders.

Combining intervention and control groups might have introduced bias. The rate of provision of follow-up samples by baseline chlamydia positives was 25% (17/68) for intervention and 40% (30/75) for control women. The redetection rates also varied by trial arm: 18% (3/17) for intervention and 30% (9/30) for control women. A higher response among chlamydia positives in the deferred screening group may have been due to recent notification of their positive baseline result, and some of this group may have used the repeat swab to check their current status before going for testing which might explain the higher redetection rate. Despite this, these groups may be reasonably similar as they were randomised from the same population, and received the same advice about attending for testing and treatment.

We had no data on possible treatment failure. We were also unable to genotype six of the 12 paired samples as four carried a *C. trachomatis* DNA load that was too low to allow genotyping, and two sets had one of the specimens used up for other studies. Thus, we had insufficient data to provide estimates of the proportion of women with redetected infection who had persistent infection versus reinfection. If the redetections were mainly reinfections, then the redetection rate of 18.5 per 100 person-years should be considered an underestimate, as we used the full follow-up time to return of the second swab in the calculation of this rate, but reinfection would have occurred earlier than this. The number of redetected infections was small, which limited the investigation of potential risk factors. Finally, as with all studies with a convenience sampling design, the generalisability of the results is limited. Participants may not be representative, and taking part in the trial and being educated about chlamydia may have influenced their behaviour.

### Comparison with other studies

Three other UK studies have examined incident and redetection rates of chlamydia among under 25 year olds eligible for the NCSP. Rates of retesting were lower than in our study and nearly all participants were attending healthcare settings.^4–6^ Lamontagne *et al* found chlamydia incidence rates in women varied between 4.6 and 10.6 per 100 person-years depending on whether participants were recruited from a general practice (GP) or a genitourinary medicine (GUM) clinic.^5^ As in our study, incidence rates were also highest among the younger age groups and those reporting new sexual partners. Redetection rates ranged from 21.1 to 29.9 per 100 person-years in GUM and GP settings, respectively.

Although population and register based studies in slightly older women found lower redetection rates,^19^ ^20^ a recent primary care based cohort of Australian women aged ≤25 years found incidence and redetection rates of 4.4% and 22.3%^17^ which are similar to the findings of ours and others.^4^ ^6^ ^21^ Few studies had data on smoking and co-infections.

### Implications

These data suggest that the annual incidence and redetection rates of chlamydia infection in women in the community are high, particularly among sexually active teenagers. They highlight the need for targeted screening among those with a new partner or recent history of infection. The recent change to the guidelines^22^ supporting routine retesting of positives needs to be publicised. A modelling study by Heijne *et al*^23^ suggests a window of 2–5 months, which is in line with retesting policies in other countries.^24–26^

**Key messages**

- The annual incidence of chlamydia in sexually active female students was 3.4 per 100 person-years, but 6.6 per 100 person-years in teenagers.
- Incident chlamydia infection was independently associated with a new sexual partner, smoking, bacterial vaginosis, high risk human papillomavirus and age <20 years.
- One in four women with chlamydia at baseline tested positive again after a median of 16 months.
- Recommendations from chlamydia screening programmes to routinely retest those who have been recently treated for chlamydia should be publicised.
